# Loss of RUNX1 function results in enhanced granulocyte-colony-stimulating factor-mediated mobilization

**DOI:** 10.1038/bcj.2016.20

**Published:** 2016-03-25

**Authors:** K Lam, A Muselman, R Du, M Yan, S Matsuura, D-E Zhang

**Affiliations:** 1 Moores UCSD Cancer Center, University of California, San Diego, La Jolla, CA, USA; 2 Divison of Biological Sciences, University of California, San Diego, La Jolla, CA, USA; 3 Department of Pathology, University of California, San Diego, La Jolla, CA, USA

RUNX1 is a transcription factor that regulates many essential aspects of hematopoiesis. Hence, disruption of normal RUNX1 function by mutations or chromosomal translocations has been found in a variety of human diseases including leukemia, myelodysplastic syndrome (MDS), myeloproliferative neoplasms and other blood malignancies. Hematopoietic stem cells (HSCs) rely on RUNX1 for their initial specification, decision to differentiate and overall homeostasis.^1, 2^ From the recently published study by Chin *et al.*,^3^ the authors reported that RUNX1 haploinsufficiency results in granulocyte-colony stimulating factor (G-CSF) hypersensitivity. Hence, HSCs have now also been shown to rely on RUNX1 for proper interaction with the bone marrow niche.^3^ Here we provide complimentary evidence that RUNX1 may mediate cell-to-cell interactions through analysis of differential gene expression and RUNX1 genome occupancy data. Furthermore, loss of normal RUNX1 function results in enhanced mobilization upon G-CSF treatment, and even more mobilization upon a combination regimen of G-CSF and AMD3100, a CXCR4 inhibitor. These findings suggest that RUNX1 mutation status should be evaluated before treatment with HSC mobilization reagents.

*Runx1* conditional knockout (KO) mice have served as a useful tool to study how disruption of RUNX1 affects hematopoiesis, as *Runx1* deletion in adults is not lethal and results in a myeloproliferative phenotype.^1, 4^ We used Ingenuity Pathway Analysis to analyze our previously published data from differentially expressed genes in HSCs (defined as lineage−, sca1+, ckit+ or LSK) from wild-type (WT) and KO mice, and from RUNX1 chromatin immunoprecipitation coupled with deep sequencing in the mouse erythroid myeloid lymphoid (also known as EML) HSC-like cell line (Supplementary Table 1).^5^ Interestingly, the top network included terms such as ‘cell-to-cell signaling' and ‘interaction, cellular movement and immune cell trafficking' (Supplementary Table 2), which suggests that RUNX1 may have a role in mediating the interaction between HSCs and the bone marrow niche.

G-CSF is commonly used as a mobilization reagent to drive HSCs from the bone marrow into peripheral blood tissues, and is one way to assess proper interaction between stem cells and the bone marrow niche.^6, 7^ In our study, KO mice were injected with G-CSF daily for 5 days and were analyzed 1 day after the final injection. Spleens from KO mice were noticeably enlarged by gross observation and by weight (Figures 1a and b). Peripheral blood from KO mice after G-CSF treatment produced significantly more colonies than blood from WT mice (Figure 1c). The increase in HSCs in the peripheral blood tissues was further confirmed by measuring the frequencies of LSK cells in the bone marrow, spleen and peripheral blood (Figures 1d–f). While comparing the two groups of mice, more LSK cells were found in the spleen and significantly more in the peripheral blood in KO mice. Notably, significantly fewer LSK cells were found in the bone marrow. These results suggest that loss of RUNX1 in the adult setting leads to hypersensitivity to G-CSF treatment and more mobilization of HSCs.

To further confirm that loss of RUNX1 function results in hypersensitivity to G-CSF, we used a previously described model that utilizes expression of a dominant-negative regulator of RUNX proteins consisting of amino acids 41–214 of RUNX1, hereafter referred to as short form (SF).^8^ RUNX1SF resembles a C-terminal truncated form of RUNX1 and includes primarily the runt homology domain, which is common among RUNX family members. Hence, RUNX1SF is believed to be a dominant-negative regulator of not only RUNX1 but all endogenous RUNX proteins. Mice transplanted with cells expressing RUNX1SF display many of the same phenotypes as KO mice but to an even more marked degree. When RUNX1SF mice were treated with the G-CSF regimen, HSC mobilization was even more pronounced as evidenced by a significant increase in LSK cells found in the spleen and peripheral blood (Figures 1g and h). In the spleen, RUNX1SF mice had an average frequency of 2.38% LSK cells compared with control MigR1 mice, which had an average frequency of 0.21%. In the peripheral blood, RUNX1SF mice displayed 1.03% LSK cells compared with 0.02% for MigR1 mice. Interestingly, in contrast to what was observed in KO mice, RUNX1SF did not exhibit a decrease in LSK cells in the bone marrow (Figure 1i). This result may be due to a heighted proliferative response to G-CSF in combination with enhanced proliferation in bone marrow LSK cells that is typically observed in RUNX1SF mice. Methylcellulose colony assays using the peripheral blood from mobilized animals confirmed that there were significantly more mobilized progenitors in RUNX1SF-transplanted mice (data not shown).

C-X-C chemokine receptor type 4 (CXCR4) is the chemokine receptor for stromal-derived factor 1 (SDF1). This receptor and ligand pair mediates the homing of HSCs to their bone marrow niche.^7^ Downregulating this signaling axis has been implicated as one of the mechanisms of how G-CSF induces mobilization of HSCs.^6, 9^ RUNX1 has also been suggested to act as a transcriptional activator of *Cxcr4*.^10^ Hence, at steady state, RUNX1 KO HSCs express lower levels of CXCR4 and are more frequently found in the peripheral circulation. Lower expression of CXCR4 may also offer one explanation for why KO mice may be more sensitive to G-CSF mobilization. To test this hypothesis, WT and KO mice were treated with a regimen of G-CSF injections followed by one injection of AMD3100, a CXCR4 antagonist also known as plerixafor. This treatment protocol typically results in a synergistic effect on mobilization when used in combination with G-CSF.^11^ KO mice displayed even more mobilization of LSK cells into the spleen and peripheral blood using the combination regimen (Figures 2a and b). The frequency of LSK cells in the bone marrow was also significantly lower after treatment (Figure 2c). The increase in mobilized HSCs was further confirmed by peripheral blood colony assay, which demonstrated more colonies from KO mice compared with WT (data not shown). When a combined G-CSF and AMD3100 regimen was used, hypersensitivity to mobilization was still observed in KO mice. The combination regimen also resulted in better mobilization compared with using G-CSF alone, suggesting that although CXCR4 has been shown to be downregulated in the absence of RUNX1, KO mice are still capable of mobilization when treated with a CXCR4 antagonist. One explanation may be that CXCR4 levels in KO mice, while lower compared with WT mice, are still expressed in adequate numbers to allow for KO mice to respond to AMD3100. Another explanation, which is further supported by the pathway analysis discussed earlier, is that other RUNX1 target genes are likely involved in regulating the hypersensitivity to G-CSF due to the loss of RUNX1. Overall, the combination of G-CSF and AMD3100 with the loss of RUNX1 function results in a tremendous amount of stem cell mobilization into the peripheral circulation.

G-CSF has been included as a priming reagent in various chemotherapy regimens for acute myeloid leukemia (AML).^12^ In addition, G-CSF has been widely used to treat chemotherapy- or disease-induced neutropenia in patients suffering from AML and MDS.^13^ Although *RUNX1* mutations in AML have largely been described in the context of being involved in the t(8;21) translocation, *de novo* mutations in *RUNX1* have been described in normal karyotype AML.^14^ In addition, *RUNX1* mutations have been associated with a significant proportion of MDS patients.^15^ This novel finding of HSC mobilization being affected by RUNX1 functional status has broad implications in the clinical setting. *RUNX1* mutational status may possibly describe and/or predict response to chemotherapy when G-CSF is used as a priming reagent or when G-CSF is used as treatment for neutropenia. In addition, to date, the pharmacogenetics of how *RUNX1* affects mobilization has not been well described. Hence, characterization of *RUNX1* mutational status at the time of diagnosis and after subsequent chemotherapy cycles may prove beneficial in guiding additional therapies. Yet another clinical implication may lay in potential HSC donors who are not responsive to traditional mobilization regimens. Modulating endogenous RUNX1 function may provide an additional avenue for peripheral blood stem cell mobilization and collection. In summary, the work described in Chin *et al.*^3^ and our own studies expand on the role of RUNX1 as a regulator of HSC mobilization, and suggest that RUNX1 mutational and/or functional status should be evaluated when mobilization regimens are used for chemotherapy or peripheral blood stem cell collection.

## Figures and Tables

**Figure 1:**
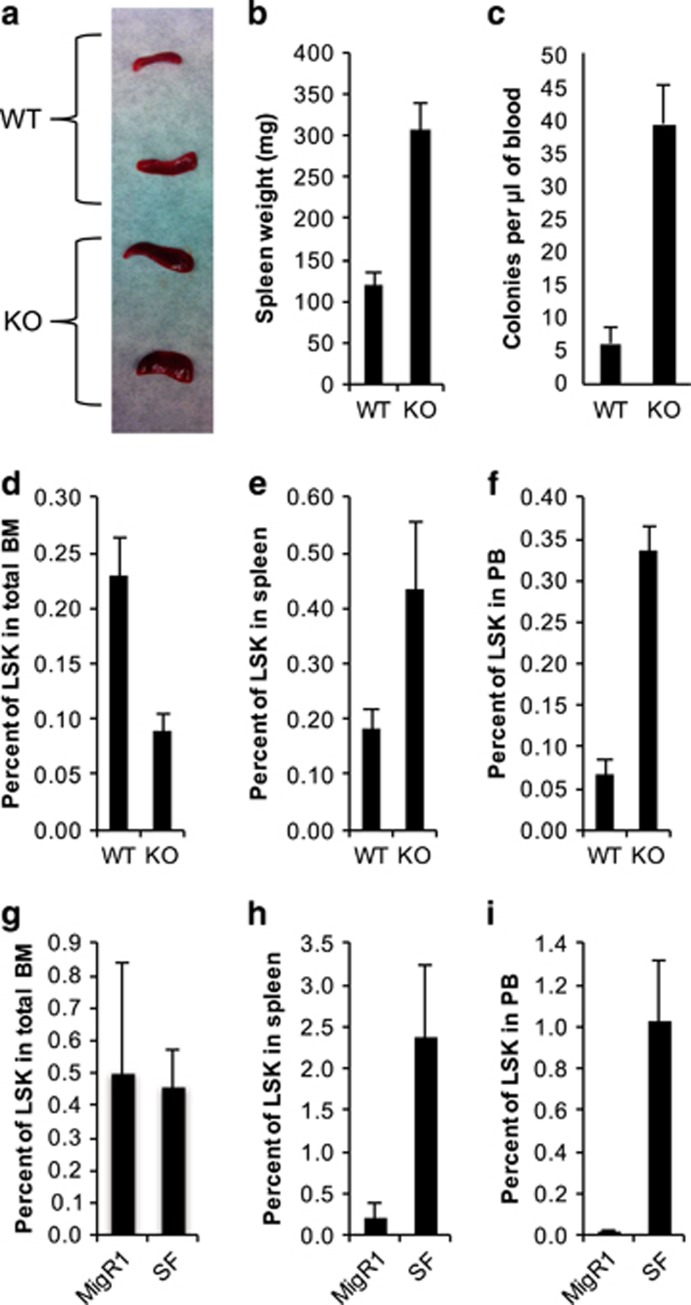
Loss of RUNX1 function results in hypersensitivity to G-CSF-induced HSC mobilization. (**a**) Representative picture of spleens from WT (top) and *Runx1* conditional KO mice (bottom) injected with a regimen of G-CSF. (**b**) Spleen weights from WT and KO mice injected with G-CSF (*n*=5 for each, 119.2 vs 306.7 mg, *P*<0.001). (**c**) Colony-forming unit assay using cells from peripheral blood of G-CSF injected mice (*n*=5 for each, WT vs KO, 6.1 vs 39.2 colonies, *P*<0.001). (**d**) Percent of LSK cells in bone marrow of G-CSF-injected mice (WT vs KO, 0.23% vs 0.09%, *P*<0.01). (**e**) Percent of LSK cells in spleens of G-CSF-injected mice (WT vs KO, 0.18% vs 0.44%, *P*=0.075). (**f**) Percent of LSK cells in peripheral blood of G-CSF-injected mice (WT vs KO, 0.07% vs 0.34%, *P*<0.001). (**g**) Percent of GFP-positive LSK cells in bone marrow of G-CSF-injected mice (*n*=4 for MigR1 vs *n*=5 for RUNX1SF, 0.50% vs 0.46%, *P*=0.87). (**h**) Percent of GFP-positive LSK cells in bone marrow of G-CSF-injected mice (MigR1 vs RUNX1SF, 0.21% vs 2.38%, *P*<0.05). (**i**) Percent of GFP-positive LSK cells in bone marrow of G-CSF-injected mice (MigR1 vs RUNX1SF, 0.02% vs 1.03%, *P*<0.01).

**Figure 2:**
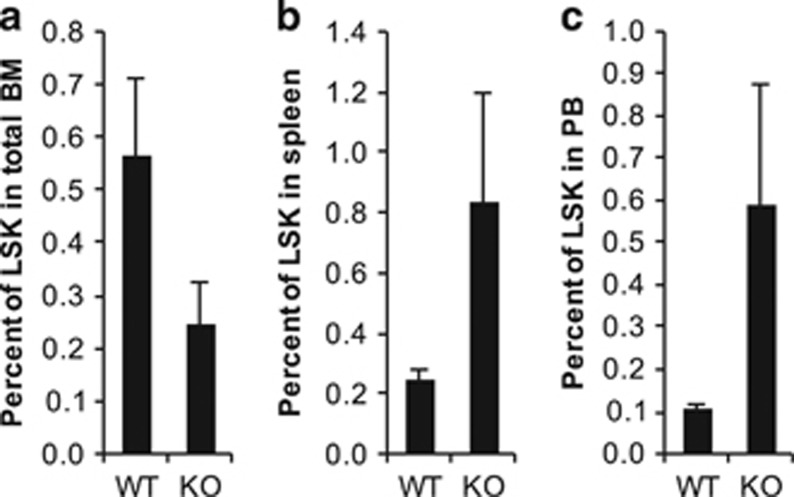
The combination of G-CSF with AMD3100 continues to have a synergistic effect on HSC mobilization in context of loss of RUNX1 function. (**a**) Percent of LSK cells in bone marrow of G-CSF and AMD3100-injected mice (*n*=5 for WT vs *n*=4 for KO, 0.56% vs 0.25%, *P*<0.05). (**b**) Percent of LSK cells in spleens of mobilized mice (WT vs KO, 0.24% vs 0.84%, *P*<0.05). (**c**) Percent of LSK cells in peripheral blood of mobilized mice (WT vs KO, 0.11% vs 0.59%, *P*<0.05).
