# Supplementary material for: Loss of RUNX1 function results in enhanced granulocyte-colony-stimulating factor-mediated mobilization
Source: Blood Cancer J. 2016 Mar 25;6(3):e407–. doi: 10.1038/bcj.2016.20 (PMC4817102; doi:10.1038/bcj.2016.20)
Supplement: Supplementary Table 1 [file bcj201620x1.pdf]

Table S1. RUNX1 target genes used for Ingenuity Pathyway Analysis.

Adamtsl4
Alcam
Atp8a2
Bdh1
Cd72
Cpa3
Csf2rb
Cyb561
Deptor
Fhdc1
Fscn1
Gzmb
Hmga2
Igf2r
Itga2b
Itga9
Jam3
Krt80
Lcp2
Map3k6
Pcdh7
Podxl
Slc24a3
Spp1
Stx3
Tjp1
Zcchc18
