# Supplementary material for: Loss of RUNX1 function results in enhanced granulocyte-colony-stimulating factor-mediated mobilization
Source: Blood Cancer J. 2016 Mar 25;6(3):e407–. doi: 10.1038/bcj.2016.20 (PMC4817102; doi:10.1038/bcj.2016.20)
Supplement: Supplementary Table 2 [file bcj201620x2.pdf]

Table S2. Top networks gathered by Ingenutiy Pathway Analysis. RUNX1 target genes are in **bold.**

Network ID	Molecules in Network	Score	Focus Molecules	Top Diseases and Functions
1	14-3-3<br>Actin<br>Akt<br>**ALCAM**<br>Ap1<br>**ATP8A2**<br>CD3<br>**CD72**<br>Creb<br>**CSF2RB**<br>**CYB561**<br>**DEPTOR**<br>ERK<br>ERK1/2<br>F Actin<br>**FSCN1**<br>**Gzmb**<br>**IGF2R**<br>IL12 (family)<br>Immunoglobulin<br>Interferon alpha<br>**ITGA9**<br>**ITGA2B**<br>**JAM3**<br>Jnk<br>**LCP2**<br>**MAP3K6**<br>NFAT (complex)<br>P38 MAPK<br>PI3K (complex)<br>Pkc(s)<br>**SPP1**<br>**STX3**<br>Tgf beta<br>**TJP1**	44	17	Cell-To-Cell Signaling and Interaction, Cellular Movement, Immune Cell Trafficking

Network ID	Molecules in Network	Score	Focus Molecules	Top Diseases and Functions
2	**ADAMTSL4**<br>ADGRF5<br>**ATP8A2**<br>**BDH1**<br>Calcineurin B<br>CALCRL<br>**CPA3**<br>DOK5<br>**FHDC1**<br>**Hmga2**<br>**KRT80**<br>Mac	28	12	Cellular Development, Connective Tissue Development and Function, Skeletal and Muscular System Development and Function

MAP3K6
Mapk
NFkB (complex)
PAQR3
PCDH7
PDE5A
PID1
Podxl
PROK1
PTGIS
RLN2
RUNX1
S100A12
SLC22A4
SLC24A3
STK10
TLR10
Tlr11
Tlr12
Tlr13
tryptase
Vegf
ZCCHC18
